# A Guide to Extract Spinal Cord for Translational Stem Cell Biology Research: Comparative Analysis of Adult Human, Porcine, and Rodent Spinal Cord Stem Cells

**DOI:** 10.3389/fnins.2020.00607

**Published:** 2020-06-18

**Authors:** Ahmad Galuta, Ryan Sandarage, Diana Ghinda, Angela M. Auriat, Suzan Chen, Jason C. S. Kwan, Eve C. Tsai

**Affiliations:** ^1^Department of Neurosciences, Faculty of Medicine, University of Ottawa, Ottawa, ON, Canada; ^2^Neuroscience Program, Ottawa Hospital Research Institute, The Ottawa Hospital, Ottawa, ON, Canada; ^3^Faculty of Medicine, The University of British Columbia, Vancouver, BC, Canada; ^4^Division of Neurosurgery, Department of Surgery, The Ottawa Hospital, Ottawa, ON, Canada; ^5^Faculty of Medicine, University of Toronto, Toronto, ON, Canada

**Keywords:** human, spinal cord, translation, neural stem/progenitor cells, proliferation, differentiation

## Abstract

Improving the clinical translation of animal-based neural stem/progenitor cell (NSPC) therapies to humans requires an understanding of intrinsic human and animal cell characteristics. We report a novel *in vitro* method to assess spinal cord NSPCs from a small (rodent) and large (porcine) animal model in comparison to human NSPCs. To extract live adult human, porcine, and rodent spinal cord tissue, we illustrate a strategy using an anterior or posterior approach that was simulated in a porcine model. The initial expansion of primary NSPCs is carried out using the neurosphere assay followed by a pharmacological treatment phase during which NSPCs derived from humans, porcines, and rodents are assessed in parallel using the same defined parameters. Using this model, NSPCs from all species demonstrated multi-lineage differentiation and self-renewal. Importantly, these methods provide conditions to enable the direct comparison of species-dependent cell behavior in response to specific exogenous signals.

## Introduction

Spinal cord injury research has relied largely on animal models to understand the mechanisms of disease and develop pre-clinical models of treatment (Cheriyan et al., 2014). Currently, there are no effective treatments for spinal cord injury which promote regeneration and restore function in humans despite numerous attempts which include physical rehabilitation, anti-inflammatory, and anti-apoptotic drugs, stem cell transplants and the use of bio-scaffolds (Varma et al., 2013; Silva et al., 2014; Badhiwala et al., 2018). The lack of successful translation from pre-clinical to patient interventions can be widely attributed to the limited understanding of biological differences between human and animal model systems, which is due to the scarcity of studies conducted with human tissue (Varma et al., 2013; Ramer et al., 2014; Silva et al., 2014; Chhabra and Sarda, 2017). Understandably, acquiring human spinal cord tissue for study is difficult for ethical and technical reasons. We report a technique of obtaining viable spinal cord tissue from neurologically deceased organ donors to enable the study of live human tissue and cell physiology. Importantly, this will also allow the direct comparison with the small and large animal models that have been advocated for translation of animal therapies to humans (Kwon et al., 2011).

One promising strategy to repair the spinal cord that has proven useful in animal studies involves the utilization of spinal cord neural stem and progenitor cells (NSPCs) (Ohori et al., 2006; Moreno-Manzano et al., 2009; Panayiotou and Malas, 2013; Kadoya et al., 2016; Yousefifard et al., 2016; Kumamaru et al., 2018). Spinal cord NSPCs are predominately located surrounding the central canal in the ependymal layer and possess the inherent ability to self-renew and differentiate into the different cell types of the central nervous system (Weiss et al., 1996; Sabelström et al., 2014). Therefore, given the correct conditions, NSPCs may be capable of regenerating the neurons, oligodendrocytes, and astrocytes that have undergone cellular death following neural insult. NSPCs may also beneficially modulate their surroundings by secreting trophic factors and providing anatomical support for cellular regeneration (Goldshmit et al., 2012; Hawryluk et al., 2012; Sabelström et al., 2013). As such, understanding the factors controlling NSPC proliferation and their differentiation is pivotal for their application in regenerative strategies. However, the majority of research into NSPC behavior has been conducted in rodent cells with limited comparison to primary human NSPCs (Dromard et al., 2008; Varghese et al., 2009; Mothe et al., 2011), thereby limiting clinical translatability. Consequently, a method to allow direct comparisons between human and animal NSPC differentiation and proliferation would enhance the clinical translation of regenerative interventions.

Rodent NSPCs have been well characterized for their differentiation and proliferation profiles using neurosphere assays, which involve the selective culture and study of NSPCs in controlled environments (Meletis et al., 2008; Hamilton et al., 2009; Kulbatski and Tator, 2009; Grégoire et al., 2015). Self-renewing NSPCs give rise to neurospheres which can be treated under defined conditions to assess the influence of exogenous signaling. NSPCs from adult human organ donors with a neurological determination of death (NDD) can be cultured in a similar manner for characterizing differentiation and self-renewal properties (Dromard et al., 2008; Varghese et al., 2009; Mothe et al., 2011). However, primary human NSPCs require an adherent basement membrane matrix such as Matrigel to expand (Mothe et al., 2011) while primary rodent NSPCs have historically been cultured as neurospheres in suspension (Weiss et al., 1996). The use of different *in vitro* models (adherent vs. suspension) impacts NSPC phenotype (Walker and Kempermann, 2014), thus a direct comparison between human and rodent NSPC studies cannot be made. Therefore, it is not clear if human and animal NSPCs differ in their differentiation and proliferation profiles.

Within this methods manuscript, we demonstrate an *in vitro* protocol to assess the intrinsic and extrinsic directed differentiation and proliferation of adult human, porcine, and rodent spinal cord NSPCs. This direct comparison of human and animal stem cell behavior is necessary to further our understanding of human NSPC regeneration potential and expands our understanding of how basic therapeutic advancements translate to clinical interventions. Human spinal cords were obtained from NDD organ donors and were processed immediately for cell culture using the neurosphere assay. NDD organ donors were identified as detailed in the Trillium Gift of Life Donor Resource Manual (Trillium Gift of Life Network, 2015). We selected porcine and rodent as our comparative species due to their predominance in previous preclinical and basic science research animal models (Cheriyan et al., 2014; Kim et al., 2018). Here, we processed primary NSPCs from humans, porcines, and rodents identically as an adherent layer to eliminate potential confounding cell culture differences and allow a direct comparison. Our culture protocol involves an expansion of primary NSPCs and allows an assessment of the proliferation and differentiation responses of human, porcine, and rodent NSPCs to extrinsic factors in parallel.

## Materials and Equipment

All procedures and experiments are performed using a sterile technique. Quantities mentioned represent the minimum requirements for the extraction and culture of one human, porcine, or rodent spinal cord.

### Spinal Cord Extraction

(1) 100 mL dissection buffer: Hank’s balanced salt solution (HBSS, Ca^2+^ and Mg^2+^ free) + 2% Penicillin-Streptomycin (PS) + 0.6% D-glucose.
(2) Tiletamine/zolazepam (Telazol^®^).
(3) Sodium pentobarbital (Fatal-Plus^®^).
(4) 50 mL falcon tube (x1; Falcon^TM^).
(5) Surgical tools for humans: Sternal saw (System 7 Sternum Saw – Stryker^TM^ 7207-000-000), bone osteotome, 32 mm, 9 ½″ (Blacksmith Surgical, BS-13-34329), mallet (Blacksmith Surgical, BS-13-34011), Deaver Retractor #3 (Sklar Surgical Instruments, 60-3212), Debakey tissue forceps (Sklar Surgical Instruments, 52-5307), Mayo scissors, straight (Sklar Surgical Instruments, 15-1555), Metzenbaum scissors (Sklar Surgical Instruments, 22-1057), Harrington-Mixter clamp (Sklar Surgical Instruments, 55-3012), scalpel handle #3 (BS-01-10001), surgical Scalpel Blade No. 10 (Bard-Parker^®^ Cat. No. 37110).
(6) Surgical tools for porcines: Autopsy saw (Stryker^TM^ Model #810 – Mopec, BD001), spinal column blade (Mopec, BD112), large section blade (Mopec, BD101), bone osteotome, 15 mm, 8″(Blacksmith Surgical, BS-13-34270), mallet (Blacksmith Surgical, BS-13-34011), scalpel handle #4 (BS-01-10003), surgical Scalpel Blade No. 23 (Swann-Morton^®^ No. 0310), scalpel handle #3 (BS-01-10001), surgical Scalpel Blade No. 10(Bard-Parker^®^ Cat. No. 37110), Adson needle holder (Blacksmith Surgical, BS-09-26025), Adson tissue forceps (Blacksmith Surgical, BS-9024), microscissors (McPherson-Vannas Micro Scissors, straight, 8 cm long, 0.1 mm tips, 5 mm blades; Kent Scientific Corporation, INS600124), paddle retractors (Kelly Retractors 64 × 76 mm, 10.5″; Medline Industries, Inc., MDS1815630), finger retractors (Finger Volkmann Retractors 6 prong, 4 1/2″; Medline Industries, Inc., MDS1838106).
(7) Surgical tools for rodents: Curved standard operating scissors (Medline Industries, Inc., MDS0812115), tissue forceps with teeth (Medline Industries, Inc., TRI66190H), fine operating with scissors straight blades (Medline Industries, Inc., MDS0800411), hemostat forceps (Medline Industries, Inc., MDS1222310), fine forceps (Medline Industries, Inc., DYND04046H), micro-dissection scissors (Medline Industries, Inc., MDG3860761).

### Central Canal Dissection

(1) 100 mm Petri dishes (x3) with 15 mL HBSS in each.
(2) 1.5 mL microcentrifuge tube (x1).
(3) Dissection microscope.
(4) Tray with ice.
(5) Surgical tools for human and porcine: fine forceps (x3) (Medline Industries, Inc., DYND04046H), scalpel handle (Medline Industries, Inc., MDS10801), scalpel blade No. 13 (Swann-Morton^®^ No. 0239), Iris scissors with straight edges (Medline Industries, Inc., MDS0859411), Wescott micro-scissors with curved blades (Medline Industries, Inc., MDS0910311).
(6) Surgical tools for rodent: fine forceps (x3) (Medline Industries, Inc., DYND04046H), scalpel handle (Medline Industries, Inc., MDS10801), scalpel blade No. 13 (Swann-Morton^®^ No. 0239), micro Iris scissors with straight edges (Medline Industries, Inc., MDS0729836), McPherson-Vannas micro Iris scissors with curved blades (Medline Industries, Inc., MDS0707764).

### Tissue Dissociation, Purification and Primary Cell Seeding

(1) Papain Dissociation kit (Worthington biochemical Inc., LK003150).
(2) Rotary Shaker.
(3) Centrifuge.
(4) 15 and 50 mL canonical tubes (Falcon^TM^).
(5) Serological pipettes (5, 10, and 25 mL, Corning^TM^).
(6) 40 μm nylon cell strainer (VWR, 10054-462).
(7) Hemocytometer.
(8) Matrigel (Growth factor reduced; Corning^TM^, 354230) coated six-well plates (see note 1).
(9) Neurobasal-A medium (Thermo Fisher Scientific, 10888022).
(10) L-glutamine, 200 mM (Gibco, A2916801).
(11) Penicillin-Streptomycin (PS), 10,000 U/mL (Gibco, 15140122).
(12) B27^TM^ minus vitamin A (B27), 50X (Gibco, 12587010).
(13) Human recombinant epidermal growth factor (EGF, Sigma, E9644).
(14) Human recombinant basic fibroblast growth factor (bFGF2, Sigma, F3685).
(15) Heparin (Sigma, H3149).
(16) 1:1DMEM/F12 (Sigma, D8900).
(17) Hormone mix: 1:1 DMEM/F12, 0.6% glucose, 3 mM NaHCO_3_, 5 mM HEPES, 25 μg/mL insulin, 100 μg/mL apo-transferrin, 10 μM putrescine, 30 nM selenium, and 20 nM progesterone in distilled water.
(18) Serum-free media (SFM): Neurobasal-A medium, 2 mM L-glutamine, 100 U/mL PS, 1% B27, and 10% hormone mix.
(19) Proliferation media (EFH): SFM + 20 ng/mL EGF + 20 ng/mL bFGF2 + 2 μg/mL heparin.

### NSPC Culture and Passaging

(1) Proliferation media (EFH).
(2) Ultra-low attachment six-well plates (Corning^TM^, C3516).
(3) StemProAccutase (Gibco, A1110501).
(4) Hemocytometer.
(5) 15 mL canonical tubes.

### NSPC Treatment Assay

(1) Proliferation media (EFH).
(2) Fetal bovine serum (FBS) (Corning^TM^, 35015CV).
(3) 96 well plate with black wells (Thermo Fisher Scientific, 137101) coated with Matrigel-growth factor reduced.
(4) 1X Phosphate buffer saline (PBS): [NaCl] = 137 mM, [KCl] = 2.7 mM, [Na_2_HPO_4_] = 10 mM, [KH_2_PO_4_] = 1.8 mM, pH 7.4.
(5) Bromodeoxyuridine (BrdU, Sigma, B5002).
(6) StemProAccutase.
(7) Hemocytometer.
(8) 15 mL canonical tubes.

### NSPC Characterization

(1) 4% paraformaldehyde (Sigma, P6148) in 1X PBS, pH 7.4.
(2) 1X PBS.
(3) Normal goat serum (NGS, Thermo Fisher Scientific, P131873).
(4) 0.3% Triton-X (Sigma, X100) in1X PBS.
(5) 2 M hydrochloric acid.
(6) Borax buffer, pH 9.2.
(7) Hoechst 33342, trihydrochloride, trihydrate – 10 mg/mL (Invitrogen, H3570).
(8) Primary antibodies: β-III tubulin (STEMCELL Technologies, 01409), GFAP (EMD Millipore, Ab5804), O4 (R&D Systems, MAB1326), Sox2 (Abcam, Ab97959), BrdU (EMD Millipore, MAB4072) (see note 2).
(9) Secondary antibodies: Goat anti-mouse IgG AlexaFlour^®^488 and 594 (Abcam, ab150113 and ab150116), Goat anti-Rabbit IgG AlexaFlour488 and 594 (Abcam, ab150077 and ab150080) (see note 2).

## Methodology

**FIGURE 1:**
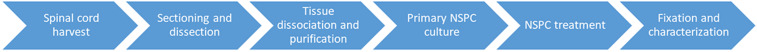
Experimental work-flow depicting spinal cord extraction (20–40 min), central canal dissection (20–40 min), tissue dissociation and purification (2–3 h), NSPC culture (up to 10 weeks), treatment (up to 3 weeks), and characterization (2 days).

This protocol, summarized in Figure 1, describes the extraction of human, porcine, and rodent spinal cord tissue using an anterior approach for humans and a posterior approach for porcines and rodents. It also describes how to culture NSPCs from all species using identical methods to allow the direct comparison of cellular behavior.

All animals were treated in strict compliance with the Canadian Council on Animal Cares guidelines for the Care and Use of Experimental Animals, all protocols were approved by the Animal Care Committee of the Ottawa Hospital Research Institute. For the extraction of human spinal cord tissue, ethics approval was obtained from the Ottawa Health Science Network Research Ethics Board. The consent form and process complied with the template provided by the University of Ottawa^1^ and followed the Tri-Council Policy Statement Guidelines (Canadian Institutes of Health Research, 2018). Informed written consent was obtained from the next of kin of the deceased organ donor.

### Spinal Cord Extraction for Humans (20–40 min), Porcines (30–40 min), and Rodents (10–20 min)

**FIGURE 2:**
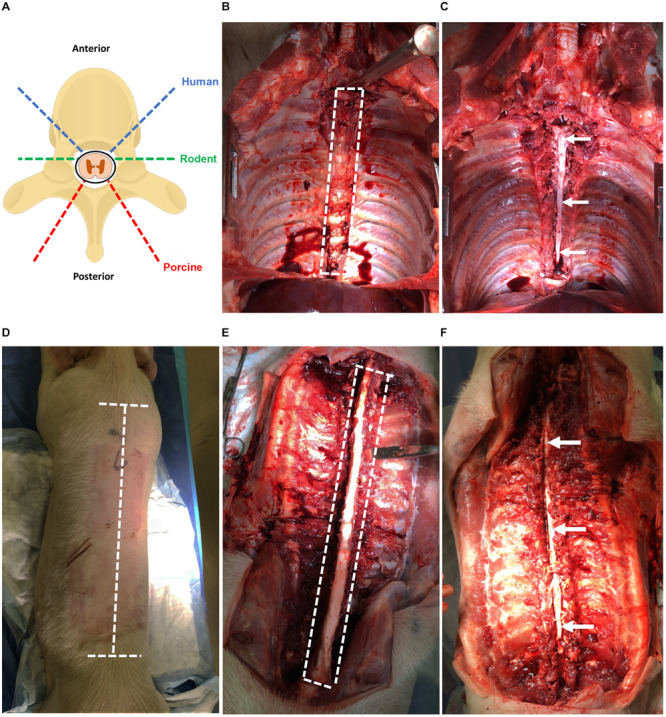
**(A)** Angles of approach for laminectomy in human, rodent, and porcine to extract the spinal cord. The dashed blue line indicates humans; green indicates rodents; red indicates porcines. **(B)** Laminectomy of the human spinal cord using anterior approach simulated in a porcine (T2–T12). Heart, lungs, esophagus, and major vessels have been removed for improved visibility. The dashed white line indicates the region of the spinal cord to be cut. **(C)** Exposed spinal cord post-laminectomy using the anterior approach from T2–T12. The thecal sac containing the spinal cord tissue is indicated by the arrow. **(D)** Laminectomy of the porcine spinal cord using a posterior approach with incisions lines marked. The dashed white line indicates incisions required to expose the vertebral column. **(E)** Laminectomy of the porcine spinal cord using the posterior approach with vertebral column exposed and paraspinal muscles removed for clarity. The dashed white line indicates cuts required to expose the spinal cord. **(F)** Exposed spinal cord post-laminectomy using the posterior approach from T1 to L2. The thecal sac containing the spinal cord tissue is indicated by the arrows.

Human spinal cord is extracted from adult (≥18 years old) NDD following aortic cross-clamping and removal of other transplant organs, approximately 2 h after cessation of circulation. If the heart and lungs are retrieved for transplant, then the rostral thoracic spinal cord is more easily attained. The spinal cord tissue is extracted using the same anterior exposure that was used for transplant organ removal (simulated in porcine, Figures 2A–C).

(1) Contain the remaining tissues and organs with a green towel and use a Deaver retractor to retract and expose the spinal column.
(2) Identify the sacral promontory and count the lumbar vertebrae to determine the L2 level.
(3) Using an osteotome and the mallet, perform a transverse wedged-shaped osteotomy through the L2 vertebral body to expose the spinal canal and allow the footplate of the sternal saw to be placed under the posterior aspect of the vertebral body.
(4) Using the sternal saw angled 45° medially (Figure 2A), cut through the vertebral bodies in a caudal to rostral direction holding the footplate of the sternal saw up against the posterior aspect of the vertebral body to ensure the cord and thecal sac are not damaged. Take care not to change the angle of the sternal saw as this could result in difficulty retrieving the sternal saw. Then, mobilize the tissues and organs to the contralateral side and perform the same maneuvers on the contralateral side.
(5) After performing the transverse cuts bilaterally with the sternal saw, remove the vertebral bodies using an osteotome to detach the rostral attachment (Figure 2C).
(6) Starting at the second lumbar vertebral level, use a closed Harrington-Mixter clamp to dissect the thecal sac circumferentially. Using the Harrington-Mixter clamp, gently retract the thecal sac ventrally. Use the Mayo or Metzenbaum scissors to transect the thecal sac at the L2 level. Then, retract the dura ventrally with forceps and use the Metzenbaum or Mayo scissors to cut the nerve roots bilaterally in a caudal to cranial direction. This allows the thecal sac containing the spinal cord to be mobilized and minimizes trauma to the spinal cord tissue itself. Once the thecal sac has been dissected to the rostral end of the vertebral exposure, transect the rostral end of the thecal sac with the spinal cord using Metzenbaum or Mayo scissors or a number 10 blade.
(7) Incise the dura with Metzenbaum scissors and place the extracted spinal cord tissue in the sterile cold (4°C) dissection media in a 50 mL conical tube on ice for transport to the tissue culture room. Typically, the spinal cord is sectioned in 20–25 cm long sections. If the thoracic organs were removed, the spinal cord can be more easily extracted to the higher thoracic levels.

While an anterior approach was used in humans because of the organ donation positioning and exposure, the extraction of porcine spinal cord described here utilizes a posterior approach given the ease of this approach and that the porcines did not undergo organ tissue retrieval and exposure.

(1) First, sedate porcines with 4 mg/kg of intramuscular tiletamine/zolazepam (Telazol^®^) and anesthetize with 2% isoflurane in 100% oxygen. Then, euthanize porcines by administrating sodium pentobarbital (Fatal-Plus^®^) intravenously at ≥100 mg/kg according to institution approved animal protocol.
(2) Orient porcine in the prone position. Using a No. 4 scalpel handle with No. 23 blade, make a longitudinal incision over the spinous processes starting from the T1 thoracic vertebrae and ending at the L2 lumbar vertebrae (see note 3). Make two transverse incisions perpendicular to the longitudinal incisions at the first thoracic vertebrae and last lumbar vertebrae (Figure 2D).
(3) Make deep incisions running adjacent to the spinous processes, lamina, and transverse processes to expose the spinous processes of the vertebrae (Figure 2E). For this cut, ensure that the incisions are deep to expose the ribs and such that all the paraspinal muscles are severed from their insertions.
(4) Expose the posterior spinal column through retraction of the paraspinal muscles with paddle or sharp prong finger retractors. If retraction is not possible, excise the para-spinal muscles and overlying skin by dissecting the fascia of the muscles using a scalpel with No. 23 blade. After the para-spinal muscles have been separated from underlying fascia, make a longitudinal incision lateral to the muscle such that the muscle can be removed.
(5) Cut through the lamina using an autopsy saw mounted with a large section or spinal column blade. Cut a through the lamina by angling the saw orthogonal to the lamina and stopping before the spinal canal (Figure 2A). Cut the vertebral column with the desired spinal cord length to be extracted.
(6) Sever the rostral and caudal ends of the spinous processes using an osteotome and mallet being cautious as to not sever the spinal cord. Remove the spinous processes and lamina using an osteotome and mallet taking care to avoid damaging the cord (Figure 2F). Then, lift the dura covered spinal cord using tooth forceps and carefully dissect rostrally. Sharply cut the spinal cord at the exposed rostral end with a No. 3 scalpel handle with No. 10 blade. Use Metzenbaum or Mayo scissors and forceps to cut the nerve roots bilaterally in a caudal to cranial direction.
(7) Place the extracted spinal cord tissue in the sterile cold (4°C) dissection media in a 50 mL conical tube on ice for transport to the tissue culture room. A 25–30 cm spinal cord section from the upper thoracic to lower lumbar region can be obtained under usual conditions. The time elapsed between euthanasia and extraction of the spinal cord is 30–40 min.

The extraction of rodent spinal cord tissue also uses a posterior approach.

(1) Anesthetize rodents with 4% isoflurane in 100% oxygen and euthanize by decapitation according to institution approved animal protocol.
(2) Place rodents on their ventral surface and sterilize skin on the dorsal surface with 70% ethanol.
(3) Excise the skin on the dorsal surface around and along the vertebral column using operating scissors.
(4) Make a bilateral incision around the vertebral column at the caudal end of the lumbar spinal cord using operating scissors and then cut the vertebral column transversely.
(5) Perform a bilateral laminectomy from the point of transection toward the rostral direction. Use dissection scissors to cleave the lamina and retract the dorsal vertebral column throughout the process until the entire spinal cord length is exposed.
(6) Gently lift the spinal cord from the caudal end using fine forceps and cleave the spinal nerves in the rostral direction. Place the extracted spinal cord tissue in the sterile cold (4°C) dissection media in a petri dish on ice.

### Dissection of the Human, Porcine, and Rodent Spinal Cord (20–40 min)

**FIGURE 3:**
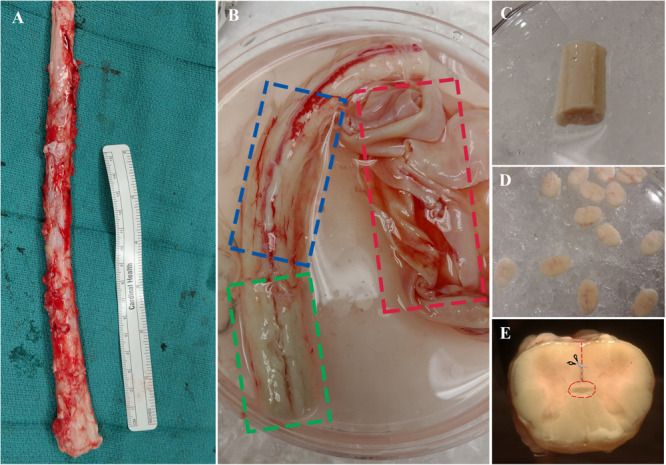
**(A)** Spinal cord is extracted from NDD adult human organ donors. **(B)** The three layers of meninges are indicated by dashed boxes; dura in red, arachnoid in blue, and pia in green. **(C)** Spinal cord segment after removal of meninges and ready for sectioning. **(D)** ∼1–2 mm thick sections to be dissected. **(E)** A cross-sectional view of the spinal cord; dashed line in red indicating direction of central canal dissection.

The procedure outlined here describes the excision of the ependymal cell containing region of the central canal of the spinal cord. This technique is novel compared to previous methods (Dromard et al., 2008; Mothe and Tator, 2015) and may be advantageous given the considerable reduction of contaminating white and gray matter. Briefly, the spinal cord is cleansed of its meninges, sectioned into thin slices (∼1–2 mm) using a scalpel blade then micro-dissected to excise a cuboidal tissue sample containing the central canal (Figure 3). Consistency in the dissection technique is necessary to obtain a similar primary cell population between biological replicates and to minimize contamination from progenitor cells in the surrounding gray and white matter tissue.

(1) Transfer the spinal cord segment using tissue forceps into the first sterile petri dish containing cold dissection buffer.
(2) Clean the spinal cord of the dura using straight-edge dissection scissors. Proceed to remove the underlying arachnoid under a dissection microscope by holding the tissue in place with fine forceps and cutting away the meninges using curved edge microscissors. Start from either end of the whole spinal cord tissue, pull away the meninges with forceps, then place one edge of microscissors between the meninges and the underlying tissue and cut all the way down to the opposite end (Figure 3B, see note 4).
(3) Once cleaning is complete (Figure 3C), wash the spinal cord in cold dissection buffer and place it in a new petri dish with cold dissection buffer for sectioning.
(4) Proceed to section the tissue into thin ∼1–2 mm sections using a scalpel blade and forceps. Start at either end of the tissue and orient the forceps downwards to hold the tissue on its lateral ends. Using the perpendicular edge of the forceps as a guide for the blade, proceed to section the tissue transversely. Sectioning is facilitated by the removal of meninges in the previous steps.
(5) Transfer the thin sections to a new petri dish with cold dissection for central canal dissection (Figure 3D). Initiate dissection from the ventral sulcus and cut toward the central canal using curved edge microscissors; use forceps to hold tissue sections in place during dissection. Stop cutting just before reaching the central canal and start cutting toward either lateral direction. Complete the dissection by cutting around the central canal region to excise a cuboidal piece (Figure 3E). It is important to use curved edge micro-scissors in this step to avoid excision of the central canal region from the underside of the section.
(6) Ensure enough spinal cord is sectioned to yield enough primary cells for culture. Typically, 5–7 sections yield 1–5 × 10^6^ cells. Alternatively, one gram of dissected central canal tissue yields 5 × 10^7^ cells.
(7) Transfer the dissected central canal tissue into sterile 1.5 mL microcentrifuge tubes and mechanically dissociate the tissue using scissors until completely minced. Keep the microcentrifuge tubes on ice while preparing the tissue dissociation kit.

### Tissue Dissociation, Purification and NSPC Seeding (2–3 h)

(1) The minced central canal tissue is dissociated into single cells using a papain / DNase treatment from Worthington Biochemicals, according to the manufacturer’s instructions. Transfer the minced tissue into a 15 mL conical tube containing the papain solution and place it on a rocker platform at 37 degrees. Use 0.2–0.4 g of tissue per vial provided in the kit and incubate for 1–2 h. This should yield 1–2 × 10^7^ cells per vial.
(2) Following digestion, triturate using a 10 mL pipette until the tissue fragments are disintegrated to form a cloudy solution.
(3) Centrifuge at 500 × *g* for 5 min, discard the supernatant, and resuspend the cells in an ovomucoid/albumin solution from the dissociation kit to inhibit papain activity.
(4) Set up a discontinuous gradient centrifugation to purify NSPCs of contaminant (post-mitotic and glial) cells and debris. Using a 10 mL pipette, gently layer the resuspended cells in step 3 onto 5 mL of albumin-ovomucoid solution from the dissociation kit.
(5) Centrifuge at 70 × *g* for 6 min, discard the supernatant, and resuspend the cells in 10 mL of warm EFH.
(6) Filter the cells through a 40 μm sterile filter using 100–1000 μL tips, add 30 mL of warm EFH, and then centrifuge at 300 × *g* for 5 min.
(7) Resuspend the cells in 1 mL of warm EFH and count the cell density using a hemocytometer.
(8) Seed the primary cells in 6-well plates pre-coated with Matrigel at a density of 20 cells/μL in a total of 4 mL EFH then incubate at 37°C, 5% CO_2_, 20% O_2_ for 1 week undisturbed.

### Feeding (15–30 min) and Passaging (30–45 min) Primary NSPC Cultures

The protocol described in this section is based on Mothe and Tator (2015), who have optimized *in vitro* culture conditions and techniques to successfully propagate adult human spinal cord NSPCs through multiple passages. They also demonstrated that human primary NSPCs require an adherent substrate to be successfully passaged, which can then be assessed as neurospheres. Rodent NSPCs do not require an adherent substrate for expansion of primary cells; but since the type of culture (adherent vs. suspension) is known to influence population phenotype, they are cultured similarly as human NSPCs.

**FIGURE 4:**
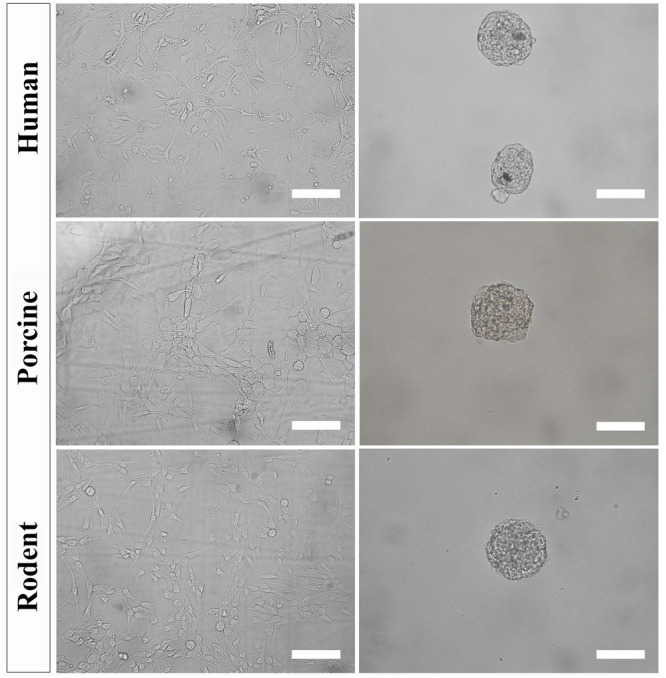
Primary NSPC cultures from adult human, porcine, and rodent spinal cord are cultured under identical conditions as an adherent layer (column 1). NSPCs can be passaged and cultured in suspension at low cell density to form neurospheres (column 2). Images were taken at 20x magnification. Scale bar = 100 μm.

(1) After 1 week, replace 50% of the medium with warm EFH containing double the concentration of mitogens (40 ng/mL EGF and FGF2). NSPCs are fed this way every 2 or 3 days for the next 1–3 weeks until adherent cultures are visibly expanding (see note 5).
(2) Once proliferating NSPCs are visible (Figure 4), remove 100% of the media, wash with warm PBS, and replace media with 2 mL of fresh warm EFH. Cultures are fed by replacing 100% of the media every 2–3 days while monitoring their growth (see note 6).
(3) Passage the cells before they reach confluence using Accutase (see note 7). Add enough Accutase to cover the surface (1 mL per well of a 6-well plate). Incubate at room temperature for 5–10 min while regularly observing to see if cells have lifted. Knock the plate on the side to detach cells.
(4) Transfer the lifted cells into a sterile conical tube, wash each well with warm PBS and transfer washes into the tube.
(5) Centrifuge at 300 × *g* for 5 min, discard the supernatant and resuspend in 1 mL of warm EFH.
(6) Count the cell density to seed NSPCs for secondary expansion or conditioned treatment.

### Generating Neurospheres (30–45 min)

To further select for self-renewing NSPCs and eradicate progenitors, cultures must be passaged and seeded in suspension. Under these conditions, self-renewing stem cells will form neurospheres that can be separated from non-stem cells by mass centrifugation. However, it is important to note that NSPC behavior changes with increased time spent in culture and away from their physiological niche. The steps below can be used to culture secondary (and beyond) neurospheres of human, porcine, and rodent NSPCs (Figure 4).

(1) Seed secondary NSPCs in 6 well plates at a density of 10 cells/μL in a total of 2 mL EFH then incubate at 37°C, 5% CO_2_, 20% O_2_.
(2) Allow NSPCs to grow for 1 week unperturbed to prevent neurosphere aggregation (see note 8).
(3) After 1 week, transfer the media containing neurospheres into a 15 mL conical flask. Wash the wells with warm PBS and transfer washes into the 15 mL conical flask.
(4) Centrifuge at 300 rpm for 5 min, discard the supernatant containing dead/single cells and resuspend in 1–2 mL Accutase. Triturate using a 1000 μL pipette tip and incubate for 5–10 min at room temperature.
(5) Centrifuge the cell suspension at 1500 rpm for 5 min, discard the supernatant, and resuspend in 1 mL warm EFH.
(6) Count the cell density and seed NSPCs for tertiary expansion (10 cells/μL) or conditioned treatment.

### Treatment and Direct Comparison of Species NSPC (Up to 3 Weeks)

Here, we propose a flexible high throughput assay to treat human, porcine, and rodent NSPCs identically and to characterize NSPC proliferation and differentiation. The parameters of this assay (cell density, culture conditions, and time spent in culture) were all optimized for the treatment of NSPCs for up to 2 weeks without becoming over confluent. Importantly, this assay is versatile to allow the assessment of various exogenous factors.

(1) Seed primary derived NSPCs onto Matrigel-coated 96-well plates in a total of 150 μL EFH. The cell density used (1–5 cells/μL) will depend on the treatment (see note 9). Leave cultures incubated at 37°C, 5% CO_2_, 20% O_2_ for 3 days undisturbed. This step is to further select for NSPCs and allow enough time for NSPCs to adhere to the basement surface.
(2) Aspirate the media and wash with warm PBS (50 μL). Ensure that conditioned media/treatments are prepared and warmed at 37°C.
(3) To induce differentiation, replace the PBS wash with 1% FBS in 150 μL SFM. To induce proliferation, replace the PBS wash with 150 μL EFH. Incubate at 37°C, 5% CO_2_, 20% O_2_ and replace media with corresponding fresh media every 2–3 days. NSPCs can be treated up to 2 weeks in 1% FBS or EFH without reaching confluency.
(4) To assess proliferation, a DNA analog (e.g., BrdU, EdU) can be used as a marker for the S-phase of the cell cycle. In our experiments, BrdU (10 μM) is added 24 h before fixing.
(5) Fix NSPC cultures at desired time points (up to 2 weeks) using 4% PFA. Incubate cultures with PFA for 20 min at room temperature, followed by three PBS (50 μL) washes. Cultures need not be characterized immediately; add a total of 100 μL PBS into each well and store the plate at 4 degrees.

### Characterization of NSPC Proliferation and Differentiation (2 Days)

A method of NSPC characterization by immunocytochemistry is proposed here. This involves the fluorescent labeling of specific cell phenotypes according to their molecular signatures (see Table 1). A working volume of 50 μL/well is used for a 96 well plate.

**TABLE 1:** Descriptions of the antibodies used for NSPC characterization including antibody specificity, dilution used, and source.

**Marker**	**Specificity**	**Dilution**	**Antibody manufacturer**
β-iii tubulin	Cytoskeletal protein abundant in neural precursors	1:500	STEMCELL Technologies
GFAP	Intermediate filament cytoskeletal protein expressed in astrocytes and brain stem cells	1:1000	EMD Millipore
O4	Membrane receptor in mature oligodendrocytes	1:500	R&D Systems
BrdU	Cell cycle (S-phase) checkpoint used as a proliferation marker	1:2000	EMD Millipore
Sox2	Transcription factor necessary for the self-renewal of neural stem cells	1:1000	Abcam

*GFAP, glial fibrillary acidic protein; BrdU, bromodeoxyuridine; Sox2, SRY-related HMG box.*

(1) Remove PBS from each well to be stained and add 50 μL of PBS for 10 min at room temperature to equilibrate.
(2) Pre-treat with 10% NGS to prevent non-specific antibody binding and with 0.3% Triton-X to permeabilize cells for 30 min at room temperature. If staining for membrane receptors, such as O4, skip the permeabilization step. Wash each well three times with PBS for 5 min at room temperature.
(3) For BrdU staining only, DNA denaturation is required by incubating cultures with 2 M HCl at 37 degrees for 30 min. Wash twice with borax for 5 min each followed by three washes with PBS for 5 min each.
(4) Dilute the primary antibody in 10% NGS in PBS according to the optimized dilution (see Table 1) and incubate at 4 degrees overnight.
(5) The next day, wash each well three times with PBS for 5 min at room temperature.
(6) Dilute the secondary antibody (1:500) in PBS and incubate for 2 h at room temperature (see note 10). Wash three times with PBS.
(7) Counterstain nuclei with Hoechst (1:2000 dilution in PBS) for 5 min at room temperature. Wash twice with PBS, add 100 μL PBS into each well for storage. NSPCs are now ready to be visualized by immunofluorescence.

## Discussion

**FIGURE 5:**
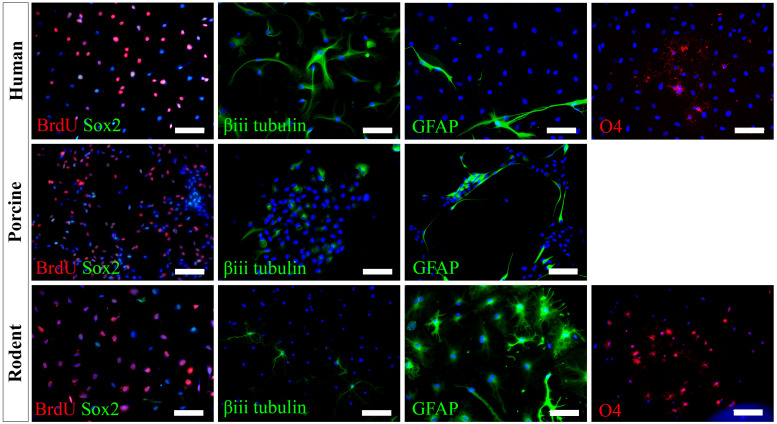
Human, porcine, and rodent primary NSPCs grew in EFH proliferate (BrdU^+^) and express neural stem cell marker Sox2. Upon differentiation, NSPCs are multipotent and generate β-iii tubulin^+^ neural precursors, GFAP^+^ astrocytes, and O4^+^ oligodendrocytes. No O4^+^ staining was observed from porcine NSPCs. Scale bar = 100 μm.

We have successfully cultured primary spinal cord NSPCs from three species using comparable dissection techniques and identical culture conditions. Here, we describe a model to treat NSPCs with exogenous factors using the same defined parameters for all species, allowing for a direct comparison of species NSPC proliferation and differentiation. We also describe a means to characterize NSPC proliferation and differentiation using immunocytochemistry (Table 1) which permits the visualization and quantification of cellular phenotypes. We confirmed that human, porcine, and rodent NSPCs are self-renewing (Figure 4) and maintain high expression of neural stem cell marker Sox2 when stimulated to proliferate with mitogen treatment (Figure 5). Unlike NSPCs in the brain that co-express Sox2 and GFAP, NSPCs in the mammalian spinal cord express Sox2 but not GFAP, allowing for the characterization of spinal cord NSPCs using Sox2 alone (Meletis et al., 2008; Becker et al., 2018). We’ve shown that human and rodent NSPCs are multipotent and differentiate into β-iii tubulin^+^ neurons, GFAP^+^ astrocytes, and O4^+^ oligodendrocytes (Figure 5 and Table 2). We also found that porcine NSPCs differed in that they did not form identifiable O4^+^ oligodendrocytes under the conditions tested. Therefore, our model, for the first time, can allow direct comparisons of human and animal NSPC proliferation and differentiation utilizing identical culture conditions so that intrinsic differences between the human and animal cells can be identified.

**TABLE 2:** Expected differentiation profile of primary-derived spinal cord NSPCs that have been treated with 1% FBS for 7 days for humans, porcines, and rodents.

	**Human**	**Porcine**	**Rodent**
Neurons	≈45%	≈20%	≈10%
Astrocytes	≈5%	≈35%	≈40%
Oligodendrocytes	<1%	*	≈5%

*^∗^ Oligodendrocytes were not detected with the O4 antibody and under the conditions tested.*

Initially, primary NSPCs from all species were stimulated to proliferate with mitogen treatment (EGF, FGF2) and grown on Matrigel, a necessity for human (Mothe et al., 2011) and porcine but not rodent NSPCs. We have cultured NSPCs from all species on Matrigel as an adherent mono-layer since the culture system (adherent vs. suspension) may affect the proportion of stem, progenitor, and mature cells in the final population (Walker and Kempermann, 2014). Also, the adherent layout results in a uniform distribution of primary derived NSPCs with minimal cell-to-cell contact allowing the direct assessment of exogenous factors. The advantage of this setting is that the causal mechanisms can be easily examined by modifying the chemical composition of the media. This model, however, presents an important limitation in the interpretation of data and inference to *in vivo* behavior where cell-to-cell contact is present.

To further select for self-renewing NSPCs, primary NSPCs can be passaged and seeded at low cell density (≤10 cells/μL) to form clonal neurospheres. These neurospheres arise from the proliferation of a single stem cell and thus may better portray the NSPC lineage profile (Narayanan et al., 2016). Secondary (and beyond) neurospheres can be dissociated into single cells and assessed similarly as primary derived NSPCs using our model. It is important to note, however, that NSPC behavior is expected to change with increased time in culture and with an increased passage. Therefore, assessment of primary NSPCs portrays *in vivo* behavior best since they spend minimal time outside their natural niche (Gil-Perotín et al., 2013).

The proposed model can serve to evaluate and compare human, porcine, and rodent NSPC responses concerning many physiological and disease processes. For example, response to physiologically and clinically relevant exogenous factors can be assessed; we can treat human and rodent NSPCs with factors that are upregulated in spinal cord injury and drive known cellular responses in rodent NSPCs (Okada et al., 2004; Kang and Kang, 2008; Moreno-Manzano et al., 2009; Lacroix et al., 2014) to determine if a similar response occurs in human NSPCs. A functional assay as such could depict causal mechanisms that would be impossible to obtain otherwise from living patients or post-mortem samples. This model can also be used to optimize a combination of growth factors to promote human NSPC survival and differentiation following transplant. Growth factors are commonly used with rodent and human NSPC transplants (Karimi-Abdolrezaee et al., 2010; Lu et al., 2012; Kadoya et al., 2016; Kumamaru et al., 2018), but potential differences in signaling mechanisms between species (Mothe et al., 2011) may hinder the translation of such treatments. As such, it is important to consider how human NSPCs would respond to exogenous factor treatments, which can be assessed *in vitro* using our proposed model and with the consideration that the factors utilized in this protocol to obtain NSPCs may also have an effect due to differences in signaling mechanisms between species. Ultimately, the evaluation of human NSPCs and comparison with animal NSPCs can clarify human NSPC response to spinal cord injury and advance the translation of regenerative strategies.

According to our knowledge, adult porcine spinal cord NSPCs have not been previously characterized for oligodendrocyte differentiation. In this study, we did not observe any oligodendrocyte differentiation from our porcine cultures using the O4 antibody and under the conditions tested. While O4 has been reported to label oligodendrocytes obtained from the brains of adult porcines (Liard et al., 2009), their protocol differed in that they obtained NSPCs from the brain rather than the spinal cord, cultured their NSPCs as neurospheres rather than in a monolayer, and utilized a different O4 antibody. It is also possible that the O4 antibody used in this study does not recognize the porcine epitope and further characterization using alternative antibodies may be beneficial. This finding highlights a challenge for conducting cross-species comparisons in which reagents that cross-react with all species being tested are needed to further understand the translational assessment of animal studies to humans. Besides, we have used NG2 and Olig2 for rodent cell labeling. However, because they are markers for early oligodendrocyte progenitors and do not exclusively label the oligodendrocyte lineage, we did not pursue further assessment in humans and porcines. We also tried CNPase and PDGFR, however, while we were able to get labeling in rodents, we were unable to get labeling in humans. Thus, in the end, since we wanted to identify terminally differentiated oligodendrocytes, and because more preclinical studies are done with rodents rather than porcines, we used O4 for labeling of oligodendrocytes.

In conclusion, the model described here is the first which allows a direct comparison of the differentiation and proliferation characteristics of primary adult human and animal spinal cord NSPCs. This assay uses the same parameters for all species, is reproducible, and allows for high-throughput testing. This model can help with the identification of species-dependent cell-intrinsic mechanisms which would be important for the translation of regenerative strategies targeting human spinal cord NSPCs.

## Notes

(1) Matrigel plates should be prepared in advance and kept at 4°C until ∼1 h before use. Use a dilution of 1:25 in SFM to coat a thin Matrigel basement layer. We recommend warming the Matrigel plates at room temperature immediately after papain digestion is initiated. Matrigel polymerizes slowly at room temperature to form a basement membrane and is required for the expansion of primary human primary NSPCs. Matrigel can be replaced by other surface coatings such as laminin, poly-D-lysine, and collagen (Mothe et al., 2011).
(2) Optimization of antibody dilutions (primary and secondary) is necessary before immunostaining, especially if antibodies are purchased from a different manufacturer or lot number.
(3) Generally, the first thoracic vertebrae are the first palpable spinous process at the rostral end and the last lumbar vertebrae are the last palpable spinous process at the caudal end. There are 14 thoracic and 6 lumbar vertebrae in porcines.
(4) The spinal cord must be removed of its meninges (dura and arachnoid) to facilitate sectioning of the spinal cord into thin segments. The dura is relatively easy to remove and does not require a dissection microscope while removal of the arachnoid requires closer attention and should be performed under a dissection microscope. The spinal cord with the dura and arachnoid removed will also minimize endothelial contamination.
(5) It is important to observe cultures regularly and track any areas with cell growth. Human NSPCs start to proliferate in small patches and grow radially outward. However, cell growth is not uniformly distributed throughout the wells and is not consistent between human cultures. Therefore, each primary human culture must be frequently observed and treated uniquely.
(6) Do not allow cells to reach confluence (over-crowding) as it becomes difficult to lift NSPCs during the passage. At this stage (near confluence), we recommend having your NSPC treatment protocol and reagents ready.
(7) Accutase is a milder dissociation enzyme than trypsin and is preferred for the delicate treatment of NSPCs.
(8) It is recommended to seed NSPCs at a low cell density and expand for 1 week to minimize sphere aggregation. This would render results more variable and reduce cell viability.
(9) The cell density used depends on the conditions. For example, EFH will stimulate NSPC self-renewal while FBS will induce differentiation. The latter process involves rapid division of progenitor cells and thus NSPCs will attain confluence quicker in FBS than NSPCs stimulated to self-renewal. For this reason, it is recommended to seed NSPCs at a lower cell density (1 cell/μL) when stimulating differentiation compared to when stimulating self-renewal (5 cells/μL).
(10) Co-staining using several antibodies is possible, if all primary antibodies are generated in different animals and all secondary antibodies bear different fluorophores.

## Data Availability Statement

The datasets generated for this study are available on request to the corresponding author.

## Ethics Statement

The animal study was reviewed and approved by the Animal Care Committee of the Ottawa Hospital Research Institute. Written informed consent was obtained from the owners for the participation of their animals in this study. The studies involving human participants were reviewed and approved by Ottawa Health Science Network Research Ethics Board. Written informed consent to participate in this study was provided by the participants’ legal guardian/next of kin.

## Author Contributions

AG, ET, DG, RS, JK, and SC contributed to the design and conception. AG, RS, and JK contributed to the acquisition of data. AG implemented the study. All authors contributed to the drafting of the manuscript.

## Conflict of Interest

The authors declare that the research was conducted in the absence of any commercial or financial relationships that could be construed as a potential conflict of interest.

## Footnotes

1 “Consent Process and Templates,” University of Ottawa, accessed November 13, 2019, https://research.uottawa.ca/ethics/guidelines/consent-process
